# Hymenopteran parasitoids of the ant-eating spider *Zodarion styliferum* (Simon) (Araneae, Zodariidae)

**DOI:** 10.3897/zookeys.262.3857

**Published:** 2013-02-01

**Authors:** Stanislav Korenko, Stefan Schmidt, Martin Schwarz, Gary A.P. Gibson, Stano Pekár

**Affiliations:** 1 Department of Agroecology and Biometeorology, Faculty of Agrobiology, Food and Natural Resources, Czech University of Life Sciences, Kamýcká 129, 165 21 Prague 6, Suchdol, Czech Republic; 2 Zoologische Staatssammlung, Münchhausenstr. 21, 81247 Munich, Germany; 3 Eben 21, 4202 Kirchschlag, Austria; 4 Agriculture and Agri-Food Canada, Canadian National Collection of Insects, K. W. Neatby Bldg., 960 Carling Avenue, Ottawa, Ontario, Canada, K1A 0C6; 5 Department of Botany and Zoology, Faculty of Sciences, Masaryk University, Kotlářská 2, 611 37 Brno, Czech Republic

**Keywords:** Ectoparasitoid, host, *Calymmochilus*, *Gelis*, larva, pupa, male description

## Abstract

*Calymmochilus dispar* Bouček & Andriescu (Hymenoptera, Eupelmidae) and *Gelis apterus* (Pontoppidan) (Hymenoptera, Ichneumonidae) are newly recorded as parasitoids of the ant-eating spider *Zodarion styliferum* (Simon) (Araneae, Zodariidae). The larvae of both parasitoid species fed on juvenile spiders. The final instar larva and pupa of *Calymmochilus dispar* and the male of *Gelis apterus* are described for the first time. Both species represent new distribution records for Portugal. The biology and host associations of the parasitoids are discussed.

## Introduction

Several groups of Hymenoptera develop on spider hosts, their larvae either feeding on the spider or on its eggs (Fitton et al. 1987). Species known to use spiders as hosts include those of Ichneumonidae (Ichneumonoidea), Eulophidae, Eupelmidae, Eurytomidae and Pteromalidae (Chalcidoidea), Diapriidae (Diaprioidea), Scelioninae (Platygastroidea, Platygastridae), Pompilidae (Vespoidea), and Sphecidae (Sphecoidea) (Aubert 1969; Fitton et al. 1987; Noyes 2012). The parasitoids attack a number of spiders ranging from ground dwelling and fast moving hunters like wolf spiders of the family Lycosidae (Kessler and Fokkinga 1973) to web spiders such as orb-web weavers of the family Araneidae (Gonzaga and Sobczak 2011) that stay on webs during most of their life. Some parasitoids parasitize a wide range of spider species whereas others are narrow specialists of a single species (Fitton et al. 1987). The parasitoids and predators include solitary species or those that develop in small broods feeding in cocooned spider egg masses to endoparasitoids that develop individually within eggs, and from external koinobiont parasitoids of mobile spiders to idiobionts that paralyse one (Pompilidae) or more (Sphecidae) spiders as prey (Austin 1985; Eberhard 1970; Gauld and Dubois 2006).

**Figure 1.**
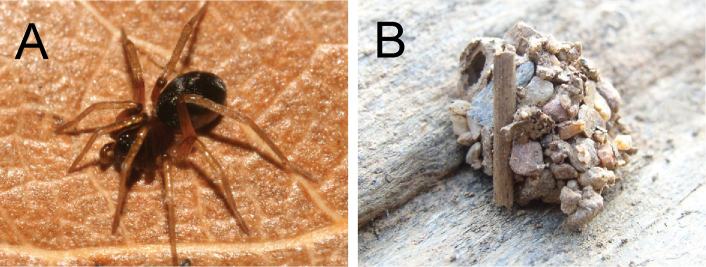
Spider host, juvenile *Zodarion styliferum* (**A**), igloo-shaped retreat (**B**).

*Zodarion* Walckenaer is the most species-rich genus of ant-eating spiders in the family Zodariidae Pickard-Cambridge (Araneae) (Platnick 2012). They are restricted almost exclusively to the Palearctic region with at least 35 species reported for the Iberian Peninsula (Platnick 2012). Available data on their biology show that all species are compulsory ant eaters (Wiehle 1928; Cushing and Santangelo 2002; Pekár 2004; Pekár et al. 2005a,b, 2011). Some *Zodarion* spiders (Fig. 1A) are Batesian mimics with various colour patterns and morphological resemblance to ants. They are crepuscular and often nocturnal wanderers. During the day they remain hidden in igloo-shaped retreats (Fig. 1B) that are attached to the underside of rocks or dead wood. The igloos provide protection against unfavourable environmental conditions and enemies such as ants.

The only previous record of a predator or a parasitoid of a *Zodarion* spider is that of a larva tentatively identified as a parasitoid feeding on *Zodarion cyrenaicum* Denis, 1935 in Israel (Pekár et al. 2005a). Here we newly present information about two parasitoids associated with *Zodarion styliferum* (Simon, 1870) in Portugal, *Calymmochilus dispar* Bouček & Andriescu (Chalcidoidea, Eupelmidae) and *Gelis apterus* (Pontoppidan) (Ichneumonoidea, Ichneumonidae). Notes on their biology are provided and the final instar larva and pupa of *Calymmochilus dispar* and the male of *Gelis apterus* are described for the first time.

## Methods

During 2008–2011 we conducted 31 field excursions in 18 localities in Central and Southern Portugal, in early spring between the last week of March and the first week of April in 2008–2010, and in late spring in the last week of May in 2011. Different habitats were investigated, including arid meadows with sparse vegetation, meadows surrounding a castle, sand beaches with sparse vegetation, slopes of river banks, olive groves, and wooded habitats. Larvae and pupae of hymenopteran parasitoids were collected from the igloos of *Zodarion styliferum* in seven localities (Fig. 2). Entomological pincers were used to open the spider igloos and parasitized spiders were transferred to plastic containers using an aspirator. The whole igloo was collected if there was a pupa inside an igloo.

**Figure:**
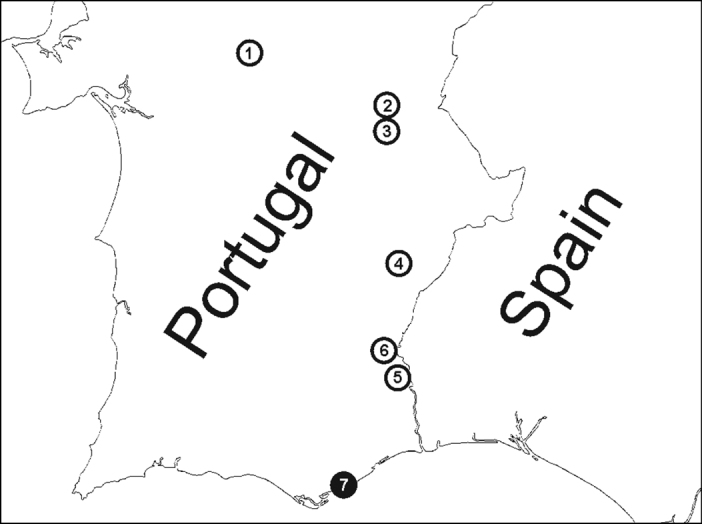
**Figure 2.** Localities where parasitoids were recovered from *Zodarion styliferum*. White circle = spiders parasitized by *Gelis apterus*, black circle = spiders parasitized by *Calymmochilus dispar*. Localities, in order from north to south: **1** Montemor o Novo – surrounding of castle ruins **2** Monsaraz – old olive grove **3** Alqueva – bank of water reservoir close to Moura **4** Ribeira de Limas – arid habitat with sparse vegetation **5** Alcoutim – arid habitat with sparse vegetation **6** Mesquita - arid habitat with sparse vegetation **7** Moncarapacho – arid slope in road surrounding.

The diversity of *Zodarion* spiders, their abundance, and the rate of the parasitism was recorded for each of the localities where *Gelis apterus* and *Calymmochilus dispar* parasitized spiders. The rate of parasitism per excursion was calculated as the number of parasitized spiders compared to all observed spiders during the excursion. Parasitoid larvae and pupae were reared until emergence of the adult wasps (1 of the 11 specimens was lost). Duration of the pupal stage, size of the pupa, and sex of the adult wasp were recorded. Emerged wasps were preserved in pure ethanol and identified using Bouček and Andriescu (1967), Gibson (1995), and Schwarz (1995, 1998 and 2002). Spiders were identified using Pekár and Cardoso (2005). The juvenile *Zodarion* hosts were identified to species-level based on knowledge of the species diversity in the investigated localities and using distinct differences in body proportions and coloration among occurring species. Wasp specimens are deposited in the private collection of M. Schwarz, Eben, Austria, the Canadian National Collection of Insects (CNCI), Ottawa, Canada, and the collection of Zoologische Staatssammlung, Münchhausenstr (ZSM), Munich, Germany. Morphological terms largely follow Gibson (1997) for Chalcidoidea and Schwarz (1998, 2002) for Ichneumonidae. The mature female and male larva and pupa of *Calymmochilus dispar* were described based on photographs taken once they were detached from the spider at two day intervals and reared to adults.

**Figure 3.**
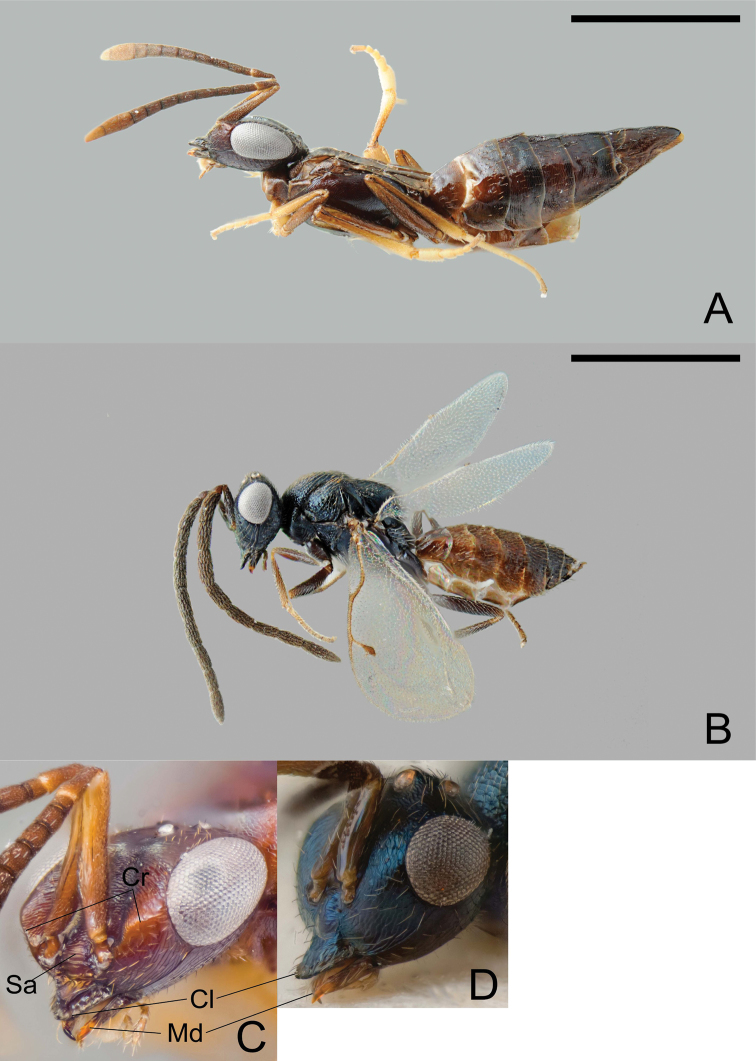
*Calymmochilus dispar*, female in lateral view (**A**), head (**C**); male in lateral view (**B**), head (**D**). Abbreviations: **Cl**: clypeus; **Cr**: crest; **Sa**: supraclypeal area; **Md**: mandible. Scale = 1 mm.

**Figure 4.**
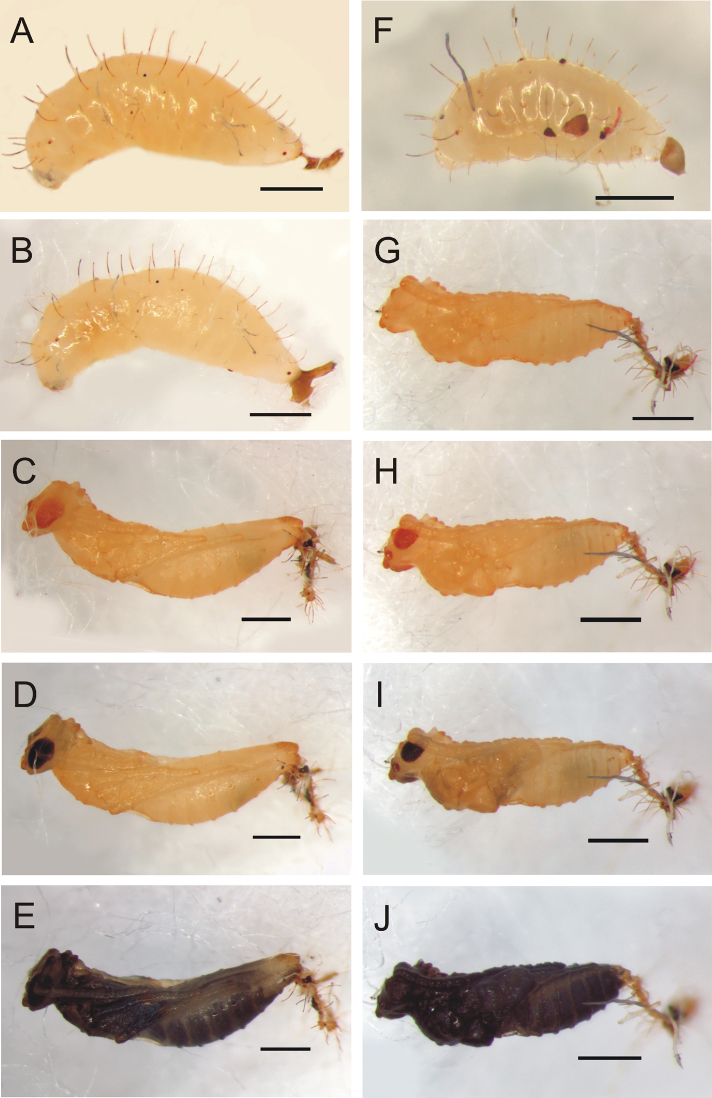
*Calymmochilus dispar*, mature larva (**A, B, F**) and pupa (**C–E, G–J**). Female final instar larva (**A, B**); female pupa after two days (**C**), six days (**D**), nine days (**E**). Male final instar larva (**F**). Male pupa after one day (**G**), four days (**H**), six days (**I**), nine days (**J**). Scale = 1 mm.

**Figure 5.**
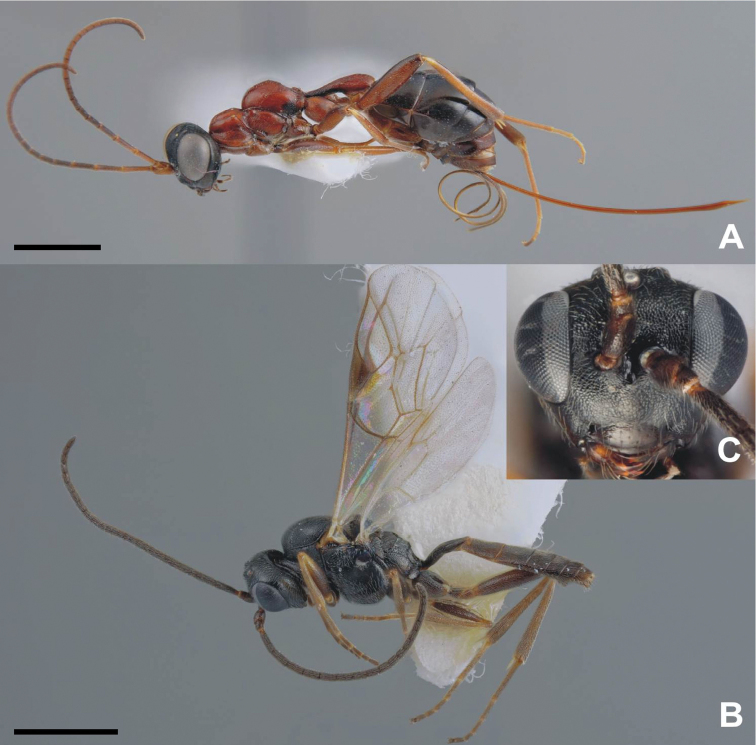
Adult of *Gelis apterus*, female in lateral view (**A**), male in lateral view (**B**), and male head in front view (**C**). Scale = 1 mm.

**Figure 6.**
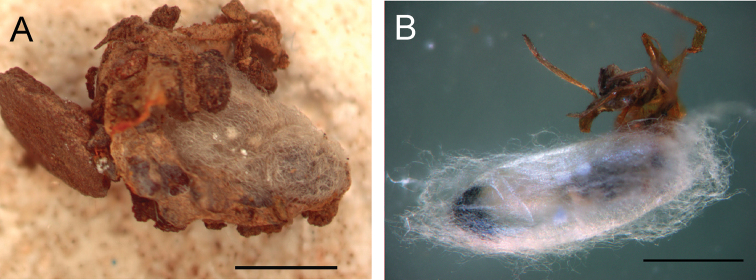
Pupa of *Gelis apterus*, inside the spider igloo (**A**) during pupation when parasitized spider was removed from igloo (**B**). Scale = 2 mm.

Microphotographs of adult wasps (Figs 3, 5) were obtained using a Nikon D300s DSLR camera with a Leitz Photar 1:2/25 mm lens connected via a Novoflex Universal Bellows (total views) and a ProgRes SpeedXT core 5 (Jenoptik AG) camera attached to Leitz M205 stereo microscope (images of heads). Images were captured in raw format, developed using Adobe Lightroom 3, and extended depth-of-field images obtained using Zerene Stacker 1.04 (Zerene Systems LLC). Stacked images were enhanced using Adobe Photoshop CS5 (Adobe Systems Inc.). The microphotographs of the cocoons, larvae and pupae (Figs 4, 6) were obtained using an Olympus U-TV 0.5 XC-3 camera with ColorView Soft Imaging System III-U software attached to an Olympus SZX-ILLK200 microscope.

## Results

### Host and parasitism rate

Three species of *Zodarion* were recorded from seven localities where parasitoids were found (Fig. 2). *Zodarion styliferum* (Simon, 1870) was dominant in all localities, whereas *Zodarion alacre* (Simon, 1870) and *Zodarion atlanticum* Pekár & Cardoso, 2005 were collected rarely. Parasitoids were found only in the igloos of *Zodarion styliferum*, which were parasitized, by the larvae of one species of Eupelmidae, *Calymmochilus dispar*,and one species of Ichneumonidae, *Gelis apterus*. This represents the first host records for these two species and new distribution records for Portugal. *Calymmochilus dispar* was recovered from only 1 of the 18 surveyed localities (Fig. 2, locality 7) where 2 of 10 examined igloos of *Zodarion styliferum* were parasitized. *Gelis apterus*was recovered from 6 of the 18 localities surveyed (Fig. 2, locality 1 – 6), with an average parasitism rate of 7% (min – max = 3% – 13%, SD = 0.03).

## Species survey

### Hymenoptera: Eupelmidae (Eupelminae)

#### Calymmochilus dispar

Bouček & Andriescu, 1967

http://species-id.net/wiki/Calymmochilus_dispar

Figures 3A–D 4A–J

Calymmochilus dispar Bouček & Andriescu (1967): 233–238. Holotype female, Romania, Agigea, 28.vii.1964, A. Andriescu (not examined).

##### Recognition.

*Calymmochilus dispar* is the only one of four European species of *Calymmochilus* (Noyes 2012) with brachypterous females. Bouček and Andriescu (1967) provided a detailed description of both sexes of *Calymmochilus dispar* in German, which are summarized below. The descriptions of the larval and pupal stages are new.

##### Description.

**Female** (Fig. 3 A, C). Length 3.0–4.6 mm. Body mostly brown to black, but partly with greenish or bluish metallic lustre, particularly frontovertex; antenna brown, clava yellowish-brown; legs brown with apices of tibiae and tarsi except for apices yellowish-brown. Head (Fig. 3C) slightly broader than mesosoma. Supraclypeal area (Fig. 3C: Sa) with about five transverse carinae and glabrous interspaces, strongly inclined from clypeus, hence clypeus below face level. Clypeus (Fig. 3 C: Cl) protruding over the mandibles, with a strongly elevated carina and a serrate margin. Lower face with a blunt crest extending from clypeus almost to ventral margin of eye (Fig. 3C: Cr). Mandible (Fig. 3C: Md) very slender, sickle-shaped. Antenna long and slender, all funicular segments longer than broad, anellus about 1.3× as long as broad, clava as long as 3.5 apical funicular segments. Mesonotum dorsoventrally compressed, with alutaceous surface sculpture. Mesoscutum flat, on same level as scutellar-axillar complex. Axillae distinguished from scutellum by only slightly finer surface sculpture. Propodeum transverse, anteriorly with a distinct transverse carina connecting propodeal spiracles, with indistinct plicae and median carina; callar region strongly declining posteriorly. Prepectus slightly larger than tegula. Wings reduced, infuscate fore wing barely extending to base of metasoma (Fig. 3 A). Metasoma evenly tapered posteriorly, syntergum tapered with rounded apex, laterally curved over to conceal very slightly exserted ovipositor sheaths.

**Male** (Fig. 3B, D). Length 1.4–2.3 mm. Head and body very dark brown to black with blue metallic lustre, metasoma brown (Fig. 3B). Antenna brown. Legs brown with knees and tarsi except for apices yellowish-brown. Head (Fig. 3D) slightly broader than thorax, nearly triangular in frontal view. Structure of lower face, clypeus (Fig. 3: Cl), and mandibles (Fig. 3: Md) similar to female except crest extending from clypeus to compound eye indicated only by slight elevation. Antenna (Fig. 3B) long, each funicular segment at least twice as long as broad, anellus indistinct and hardly discernible, claval segments fused. Mesoscutum convex with distinct notauli. Wings fully developed. Scutellum strongly convex, almost parallel-sided, with strongly inclined sides. Metanotum almost vertical, dorsellum almost triangular with surrounding furrow, dorsally with sharp carina. Propodeum with distinct median carina.

**Larva** (Fig. 4A, B, F). Brownish-yellow, female length about 2.5 mm (N = 1) and male length = 1.6 mm (N = 1). Mature larva with one pair of long, strong, dorsal setae (0.25–0.3× maximal diameter of larva) on each body segment plus two pairs of smaller dorsal setae (0.8× length of longer setae), one pair between long dorsal setae of first and second segment and second pair between those of second and third segment; laterally with one pair of smaller lateral setae (0.5–0.6× length of dorsal setae) on each body segment and irregularly placed short setae. Larval head very weakly sclerotized (not easily discernible in photographs).

**Pupa** (Fig. 4C–E, G–J). Pupa brown, about 3 mm length for female (Fig. 4C) and 2.2 mm for male (Fig. 4G). Eyes and mandibles becoming dark brown (Fig. 4C, H) as part of sclerotization process after 3 days. Eyes and mandibles dark brownish-black and first dark spots appearing inside pupa (Fig. 4D, I) seven days after pupation; pupa completely dark brown (Fig. 4E, J) after nine days.

##### Material.

PORTUGAL, Faro district: 1 ♂ and 1 ♀ Moncarapacho; rocky slope near road, in spider igloos under rocks (37°05'N, 7°47'W, Fig. 2, locality 7), penultimate larvae attached to spider abdomen, 31.iii.2009, S. Korenko leg., larvae pupated 7.iv.2009 (male) and 8.iv.2009 (female), adults emerged 22.iv.2009, (1 ♂, CNCI; 1 ♀, ZSM).

##### Distribution.

Armenia, Bulgaria, Croatia, France, Germany, Italy, Serbia, Spain, Yugoslavia (Noyes 2012) and Portugal (new record).

##### Host.

Juvenile *Zodarion styliferum* with prosoma length of 0.4–0.5 mm (N = 2) (new host record).

##### Biology.

The two *Zodarion styliferum* igloos from which *Calymmochilus dispar* were reared were collected in an open, rocky habitat with sparse vegetation. When collected, a larva was attached to the abdomen of an immobilised juvenile inside the igloo. Exuviae of the previous moults were attached to the apex of the abdomen of the last instar. The larvae did not build any cocoon inside the igloo, being protected only by their long setae. The final instar and prepupal stage combined lasted 7 days for the male and 8 days for the female, after which the larvae pupated. The female emerged 15 days and the male 16 days after pupation at 23°C (±1.5°C).

##### Remarks.

Little is known about the biology and host associations of *Calymmochilus* wasps. Previously, *Calymmochilus russoi* Gibson, 1995 was reared from olive branches infested with *Pheloeotribus scarabaeoides* (Bernard, 1788) (Coleoptera, Scolytidae) (Russo 1938) and *Calymmochilus longbottomi* Gibson, 1998 was reared from *Synsphyronus lathrius* Harvey, 1987 (Pseudoscorpionidae, Garypidae) (Austin et al. 1998). The *Zodarion styliferum* host was of a similar body size and created structurally similar igloos as the pseudoscorpion documented by Austin et al. (1998). Larvae of *Calymmochilus dispar* do not create a cocoon for pupation; rather they use the already built spider igloo to help protect the bare larvae, which is isolated from the inner surface of the igloo by their long dorsal setae. The larvae we reared from the two *Zodarion styliferum* igloos were on the underside of a rock approximately 5 cm apart from each other. The parasitized pseudoscorpions reared by Austin et al. (1998) were also located under rocks, whereas the beetle larvae associated with *Calymmochilus russoi* were under tree bark (Russo 1938). These concealed habitats presumably provide additional shelter for the *Calymmochilus* larvae and support Bouček (1988), who suggested that *Calymmochilus* species are primarily associated with hosts in sheltered places, e.g. under bark or rocks. The unusual, protuberant clypeus that characterizes adults may be a structural adaptation to help the adults emerge and the female to access restricted spaces to parasitize new hosts. However, it remains to be shown whether *Calymmochilus dispar* is narrowly associated with *Zodarion* species or parasitizes taxonomically more diverse hosts in similar niches.

### Hymenoptera: Ichneumonidae (Cryptinae)

#### Gelis apterus

(Pontoppidan, 1763)

http://species-id.net/wiki/Gelis_apterus

Figures 5A–C 6A–B

Ichneumon apterus Pontoppidan (1763): 692–693. Holotype female, missing. Comments about description in Schwarz (1995).

##### Recognition.

Schwarz (2002) gave a key to the western Palaearctic species of *Gelis* with apterous females, and Schwarz (1995, 1998) provided a diagnosis and description of the female of *Gelis apterus*. The main diagnostic features of the female are summarised below; the descriptions of the male and pupa are new.

##### Description.

**Female** (Fig. 5A). Length 3.2–5.5 mm. Apterous. Body mostly black but base of antenna orange and thorax, propodeum and first segment of gaster varying from entirely black to nearly entirely orange; legs mainly blackish or dark brown with yellowish brown parts; tibiae white basally. Antenna with 21–25 segments; third segment (without anellus) 3.7–4.4× as long as wide. Malar space 1.2–1.3× as long as wide. Mesoscutum in lateral view not or only weakly sloping anteriorly, with a strong or weak median longitudinal furrow. Mesopleuron with fine striation anteriorly or more rarely almost entirely striate. Metapleuron entirely granulate or more rarely partly smooth and lustrous. Hind femur 4.0–4.9× as long as wide. Ovipositor sheath 1.9–2.5× as long as hind tibia. Ovipositor curved upwards, its tip with only very weak teeth ventrally.

**Male** (Fig 5 B, C). Length 3.0–4.1 mm. Macropterous. Body mostly black but mandible with teeth reddish, palpi brown, and tegula yellowish brown or brown. Legs with coxae, trochanters, trochantelli and femora except for following black or blackish brown; fore and mid femora yellowish-brown apically; tibiae basally whitish (most distinct on hind tibia), fore tibia except basally, and mid tibia except basally and apically yellowish-brown, hind tibia except basally blackish brown; tarsi brown or blackish brown. Fore wing with pterostigma brown except white basally. Body mostly distinctly granulate and matt, without distinct punctures. Antenna with 21–23 segments; third segment (without anellus) 2.9–3.3× as long as wide; segments 11–13 with linear tyloids. Clypeus in profile evenly and rather weakly convex, smooth or weakly granulate dorsally in addition to some scattered punctures; lower margin convex and region just above lower margin depressed. Mandible rather long, its teeth of equal length, outer surface with a distinct swelling subbasally. Malar space 1.2× as long as basal width of mandible, and without a furrow. Genal carina joining oral carina behind base of mandible. Ocelli small. Head behind eyes in dorsal view moderately narrowed and distinctly convex. Pronotum without dorsomedian longitudinal ridge. Mesopleuron with fine granulation and weak rugosity, speculum and hind margin below speculum smooth. Prepectal carina present but rather weak. Propodeum of moderate length and with both transverse carinae complete and distinct; longitudinal carinae anterior to posterior transverse carina rather weak and absent basally except for lateral longitudinal carina; propodeum lustrous between transverse carinae and with longitudinal striation about as distinct as longitudinal carinae; lustrous anterior to posterior transverse carina, nearly smooth and with distinctly separated area petiolaris. Legs slender with hind femur 5.0–5.1× as long as wide. Fore wing with areolet rather small. Gaster with first segment slender, without median dorsal carinae, and with dorsolateral and ventrolateral carinae rather weak.

**Pupa** (Fig. 6A, B). Pupa brownish (becoming dark brown as part of sclerotization process), about 5.5–7 mm.

##### Material.

PORTUGAL, Beja district: 2 ♀ Ribeira de Limas, direction to Guadiana River, slope close to unpaved road (37°51'N, 7°31'W, Fig. 2, locality 4), 24.v.2011, S. Korenko leg. (1 pupa and 1 larva), larva on *Zodarion styliferum*, pupated 25.v.2011, adults emerged 5.vi.2011 and 7.iv.2011. Specimens deposited in collection of M. Schwarz.

Évora district: 1 ♂ Montemor o Novo, surrounding of castle ruins (38°38' N, 8°13'W, Fig. 2, locality 1), 10.iv.2010 (pupal stage), S. Korenko leg., adult emerged 12.iv.2010. 1 ♀ Monsaraz, olive grove (38°26' N, 7°32' W, Fig. 2, locality 2), 11.iv.2010, E. Líznarová and S. Korenko leg., larva on *Zodarion styliferum*, pupated 21.iv.2010, adult emerged 4.v.2010 (one empty cocoon and one larva that died in the laboratory were also collected from same locality). 1 ♀ and 1 ♂ Alqueva close to Moura, bank of water reservoir (38°12'N, 7°32'W, Fig. 2, locality 3), 11.iv.2010, S. Korenko leg., larvae on *Zodarion styliferum*, pupated 14.iv.2010, adults emerged 27.iv.2010 (female) and 28.iv.2010 (male), female escaped.

Faro district: 1 ♀ Casa do Canavial close to Mesquita in Guadiana Valley Natural Park (37°32'N, 7°31'W, Fig. 2, locality 6), 4.iv.2008, S. Korenko leg., larva on *Zodarion styliferum*, pupated 21.iv. 2008, emergence date not recorded. 1 ♀ and 1 ♂ Alcoutim, view terrace close to town (37°27'N, 7°28'W, Fig. 2, locality 5), 29.iii.2009, S. Korenko and S. Pekár leg., larvae on *Zodarion styliferum*, pupated 2.iv.2009 (female) and 1.iv.2009 (male), adults emerged 9.iv.2009.

##### Distribution.

South and Central Europe (Schwarz 1998) including Portugal (new record), Azerbaijan, Tajikistan (Schwarz 1998).

##### Hosts.

Juvenile spiders of *Zodarion styliferum* with prosoma length averaging 2.47 mm (N = 9, min/max = 1.6/3.2 mm) (new host record).

##### Biology

*Gelis apterus* and its spider hosts appear to be associated with open arid habitats with sparse vegetation. Females attack the host spiders in the igloo, penetrating the igloo wall with their long ovipositors (laboratory observation). Unfortunately nothing more is known about oviposition behaviour. The larva makes a cocoon inside the spider igloo before pupation (Figs. 6). Details about the sclerotization process of the pupa were not recorded because of its location in the cocoon. The cocoon consists of white to brownish weaved threads which fill space inside the spider igloo. Adults emerged 9–14 days after pupation.

##### Remarks.

Females of *Gelis apterus* reared from *Zodarion styliferum* in Portugal differ from those collected in other parts of Europe by somewhat longer ovipositor sheaths and the thorax laterally having smooth patches. In these two features they resemble the closely related species *Gelis atratus* (de Stefani, 1884), but females of *Gelis apterus* reared from *Zodarion styliferum* do not have the mesoscutum sloping downwards caudally.

The previously unknown macropterous males of *Gelis apterus* are very distinct from their apterous females. Males of *Gelis* are often unknown or unassociated with females because of the difference in aptery and because they are more difficult to distinguish in many species. The two sexes of *Gelis apterus* possess very few similar features that indicate they are conspecific and we consider the males we reared as *Gelis apterus* primarily because they were reared with females of *Gelis apterus*, and because only females of *Gelis apterus* were reared from *Zodarion styliferum*.

Diagnostic features of *Gelis apterus* males include an evenly and weakly convex, smooth or mainly smooth clypeus, long mandible and malar space, moderately narrowed and distinctly convex head, and the pattern of sculpture and carinae of the propodeum. These features enable separation of males from those of most other *Gelis* species. However, it is expected that the unknown males of *Gelis atratus* will be very similar to *Gelis apterus* and the two may not be easily distinguishable.

We found *Gelis apterus* associated with *Zodarion styliferum* in several parts of Central and South Portugal, and it seems to be widespread but in low abundance throughout the Iberian Peninsula based on the observation of three empty pupae inside *Zodarion* igloos in the Spanish provinces of Málaga and Granada (Korenko unpub.). *Gelis* is a large genus of parasitoid wasps that are worldwide in distribution but with most species in the Holarctic region. Some species are fully-winged but many are ant-like micropterous or wingless. Hosts of different species of *Gelis* include eggs or larvae or cocoons or cocoon-like structures of a wide range of holometabolous insects as well as egg sacs of spiders. Fitton et al. (1987) listed ten *Gelis* species reared from spider egg sacs of both wandering and web-building spiders. The larvae of *Gelis* species known to attack spiders have been regarded as exclusively feeding on spider eggs (Fitton et al. 1987). We did not observe this for *Gelis apterus*, which fed on juvenile spiders. Diurnally active female *Gelis apterus* presumably attack nocturnal *Zodarion* that are resting in their igloos during the day.

*Zodarion styliferum* is common in Portugal and has two overlapping generations (Pekár unpub.). Juvenile spider hosts are therefore available to *Gelis apterus* during the whole year and likely provide a highly available resource. *Gelis apterus* is documented from South – Central Europe to 70° eastern longitude. Several *Zodarion* species have overlapping distributions within this area. Their life history, behaviour, body size and igloo architecture are similar to *Zodarion styliferum* and could be potential hosts for *Gelis apterus*. Although we did not rear *Gelis apterus* from either *Zodarion alacre* or *Zodarion atlanticum*, this may simply reflect their relative rarity and low parasitism rates. Whether *Gelis apterus* is also associated with *Zodarion* species outside of the Iberian Peninsula is not known. Knowledge of the host range of *Gelis apterus* in other parts of its range is essential to confirm whether *Zodarion* spiders are their only hosts and, if not, understand how an association with spider hosts evolved.

## Supplementary Material

XML Treatment for Calymmochilus dispar

XML Treatment for Gelis apterus
